# Efficient foot motor control by Neymar’s brain

**DOI:** 10.3389/fnhum.2014.00594

**Published:** 2014-08-01

**Authors:** Eiichi Naito, Satoshi Hirose

**Affiliations:** ^1^Center for Information and Neural Networks (CiNet), National Institute of Information and Communications TechnologySuita, Japan; ^2^Graduate School of Medicine and Graduate School of Frontier Biosciences, Osaka UniversitySuita, Japan

**Keywords:** Neymar da Silva Santos Júnior, football brain, foot movement, medial-wall motor region, functional magnetic resonance imaging, efficient motor control, long-term physical training

## Abstract

How very long-term (over many years) motor skill training shapes internal motor representation remains poorly understood. We provide valuable evidence that the football brain of Neymar da Silva Santos Júnior (the Brasilian footballer) recruits very limited neural resources in the motor-cortical foot regions during foot movements. We scanned his brain activity with a 3-tesla functional magnetic resonance imaging (fMRI) while he rotated his right ankle at 1 Hz. We also scanned brain activity when three other age-controlled professional footballers, two top-athlete swimmers and one amateur footballer performed the identical task. A comparison was made between Neymar’s brain activity with that obtained from the others. We found activations in the left medial-wall foot motor regions during the foot movements consistently across all participants. However, the size and intensity of medial-wall activity was smaller in the four professional footballers than in the three other participants, despite no difference in amount of foot movement. Surprisingly, the reduced recruitment of medial-wall foot motor regions became apparent in Neymar. His medial-wall activity was smallest among all participants with absolutely no difference in amount of foot movement. Neymar may efficiently control given foot movements probably by largely conserving motor-cortical neural resources. We discuss this possibility in terms of over-years motor skill training effect, use-dependent plasticity, and efficient motor control.

## Introduction

It is well-established that motor practice induces plastic changes in the human central motor system even in adults. For example, when people practice a novel hand motor skill, the central motor representation in the hand section of the primary motor cortex (executive locus of voluntary motor control: M1) expands when they repeat the practice for days or weeks (Karni et al., 1995; Pascual-Leone et al., 1995). Namely, Karni et al. (1995) demonstrated the expansion of M1 activation associated with the acquisition of motor skills when the local blood oxygenation level-dependent (BOLD) signal is evaluated with functional magnetic resonance imaging (fMRI). However, much longer-term (over years) training suggests a different story.

Recently, a non-human primate study revealed that the over-years training of a motor skill with a forelimb substantially decreases the ^14^C-2-deoxyglucose (2DG) uptake widely in the forelimb section of M1 even when the monkeys perform the acquired motor skill (Picard et al., 2013). 2DG uptake can be an indicator for the metabolic activity of the brain, which is tightly coupled with such hemodynamic cerebral activity as BOLD signals. Thus, a similar training effect should be observed in the BOLD signals of human participants who have performed over-years physical training, e.g., sport training.

In the present study, we focus on the motor-cortical foot regions of professional footballers and provide valuable evidence that the football brain of Neymar da Silva Santos Júnior (the Brasilian footballer) substantially reduces the recruitment of foot motor regions during foot movements. We scanned his brain activity with a 3-tesla fMRI while he rotated his right foot (ankle) at 1 Hz and compared his brain activity with that obtained from three age-controlled professional footballers, two top-athlete swimmers, and one amateur footballer. We assumed that professional footballers have trained to perform various types of foot movements, e.g., “manipulating” and kicking a ball in many different ways by controlling ankle joint, through their intensive over-years daily training. Even though top-athlete swimmers have also trained their foot movements, these are usually highly-patterned movements and less variety is required. Thus, it is very likely that professional footballers have been exposed to richer sensory-motor experiences of foot movements and their brains must have stored a variety of repertoire of foot motor skills as compared to swimmers and amateur footballers. We hypothesized that neuronal resource in motor-cortical foot regions allocated to control a given simple foot movement is smaller in professional footballers than in swimmers and amateur footballer, because previous studies have shown that musicians (pianists and keyboard players) who should have richer sensory-motor experiences and a variety of repertoire of hand/finger motor skills reduce recruitment of motor areas during finger movements compared with musically naïve control (Jäncke et al., 2000; Krings et al., 2000; Haslinger et al., 2004; Koeneke et al., 2004). In particular, we expected that reduced recruitment of motor areas could become apparent in an exceptionally skillful footballer like Neymar who could obviously perform various repertoire of foot movements. To address these questions, the participants performed the simple foot movement task and we tested the hypotheses that professional footballers, especially Naymar, could perform this movement by recruiting less amount of brain activity in the medial-wall motor-cortical foot regions.

## Materials and methods

### Participants

Seven healthy male volunteers participated in our experiment and their ages ranged from 18–32. They included 22-year-old Neymar da Silva Santos Júnior, and three other age-controlled professional footballers who play for the 2nd division in Liga Española (JS, age 19, SC, age 18, XB, age 23), two Spanish national-training-center level swimmers (MC, age 18, YL, age 22) and one amateur footballer (SH, age 32). The following are the years of their football experience (defined as when they joined football clubs): 16 for Neymar, 12 for JS, 13 for SC, 19 for XB, and 9 for SH. No such football experience was reported by the swimmers. Except XB and YL, all the participants reported that they prefer to kick and dribble with their right foot. The local ethics committee of the ALOMAR hospital in Barcelona approved this study. All participants gave written informed consent, and the experiment was carried out based on the principles and guidelines of the Declaration of Helsinki (1975). The public disclosure of Neymar’s brain activity and the use of his name solely for academic purposes were approved by Neymar’s representative.

### MRI measurement

A 3.0-T MRI scanner with a head-coil (Discovery MR750w 3.0T, General Electric, USA) provided T1-weighted anatomical images (MP-RAGE) and functional T2*-weighted echo-planar images (64 × 64 matrix; pixel size, 3.0 × 3.0 mm^2^; flip angle, 90°; echo time, 35 ms). We collected functional volumes every 3 s (TR = 3000 ms) that comprised 48, 3.0-mm thickness slices without interslice gaps. The whole brain was within the field of view (FOV, 192 × 192 mm^2^).

### Experimental procedure

In this fMRI experiment, the participants rested comfortably in a supine position in the scanner. Their heads were immobilized by cushions, and their torsos and knees were tightly wrapped with belts to fixate them to the scanner bed. They were instructed to avoid body movements except for their right foot and to close their eyes during the scanning. Auditory instructions about the initiation and cessation timings of their right foot movements were given to them through an MRI-compatible headphone.

Each participant completed two experimental sessions, each of which consisted of 8 runs. Each run lasted for 15 s, followed by 15-s resting inter-run-intervals; the first run started 30 s after the initiation of the scanning. In each run, the participants continuously rotated their right ankle either rightward or leftward in synchronization with 1-Hz metronome sounds. The rotation direction was verbally instructed 3 s prior to each run, and the directions were altered run by run (rightward run, leftward run, rightward run, and so on). Thus, the participants completed four rightward runs and four leftward runs in each session. We collected 40 functional volumes (TR = 3 s) during the foot movements in each session. For Neymar, we conducted one extra (third) session where he generated much larger movements (see red open squares in Figure 2).

**Figure 1:**
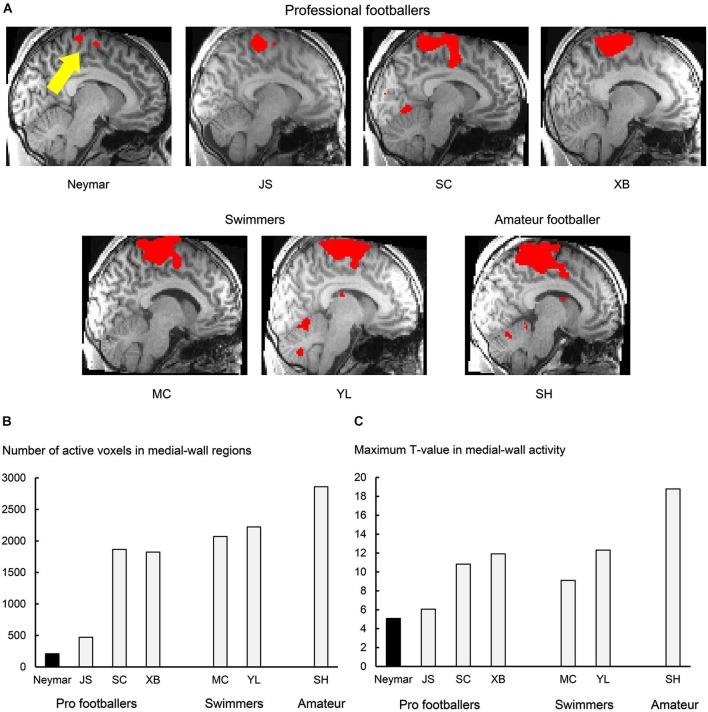
**Results from 2-session analysis. (A)** Activity in medial-wall motor regions during foot movements in each participant. Each panel displays result obtained from each participant. Voxels with activity greater than voxel-wise threshold *p* < 0.001 uncorrected (*T* = 3.12 for Neymar and *T* = 3.14 for the others) are shown in red and superimposed on an individual normalized brain. Sagittal section (*x* = −8) in left hemisphere is shown. Size of medial-wall activity was smallest in Neymar’s brain (yellow arrow). **(B)** Number of active voxels (*p* < 0.001 uncorrected) in medial-wall regions during foot movements in each participant. **(C)** Maximum *T*-value in medial-wall activity in each participant. In both panels **(B)** and **(C)**, black bars indicate Neymar’s data. Both number of active voxels and maximum *T*-value were smallest in Neymar.

**Figure 2:**
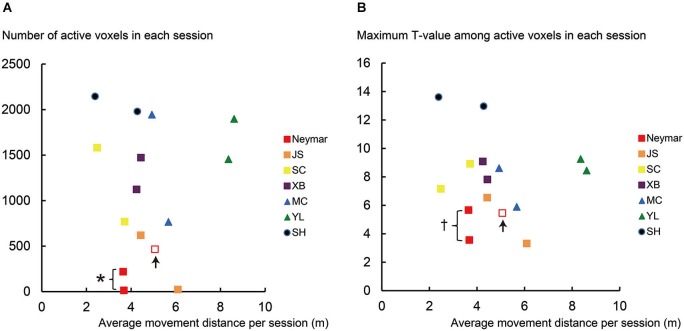
**Results from single-session analysis. (A)** Relationship between number of active voxels (y-axis) and average movement distance (x-axis) for each session of each participant. **(B)** Relationship between maximum *T*-value in active voxels (y-axis) and average movement distance (x-axis) for each session of each participant. Note that data in x-axis are identical between panels **(A)** and **(B)**. Different colors in plotted data indicate different participants. Squares indicate data obtained from professional footballers. Data obtained from an extra third session in Neymar are shown in open red squares with up arrows. Asterisk (*) in panel **(A)** means that number of active voxels in Neymar (red filled) was significantly smaller than those in remaining six participants (individual analysis with Mann-Whitney test; *p* < 0.05). Cross (†) in panel **(B)** means that his maximum *T*-value (red filled) showed significantly smaller trend than those in the remaining six participants (*p* = 0.088). Even though number of active voxels and maximum *T*-values in pro footballer JS (orange) were also small, they did not reach significant level (*U* = 3, *p* = 0.13 for both) due to Neymar’s data. In panel **(A)**, the number of active voxels plotted very close to horizontal axis was 13 for Neymar (red filled) and 22 for JS (orange). Even though the number of active voxels was smaller in these cases, this activity should have physiological importance because these voxels in each brain formed a cluster in the ROI, which was actually the only one cluster in the entire brain. And most of these voxels (10/13 voxels for Neymar and 22/22 voxels for JS) in each brain were consistently active during foot movements between the two sessions.

For each participant, we recorded the foot movements (30 frames per second) by a digital video camera (HDR-PJ760, Sony, Japan) that was located away from the end of the scanner bed (just outside the five gauss line) at approximately the same height as the bed. Thus, the movements were recorded from the view of the bottom of the foot. The bed’s width was also recorded for the calibration to quantify the foot movements (see below).

### fMRI data analysis

We analyzed the fMRI data using Statistical Parametric Mapping software (SPM5, Wellcome Department of Cognitive Neurology, London, UK) that was implemented in Matlab 2007a (MathWorks, Inc.). We excluded the first five functional images of each session from our analysis to allow for magnetization equilibrium. The remaining functional images were realigned to correct for head movements and co-registered to each participant’s anatomical image. Both the functional and anatomical images were normalized to the Montreal Neurological Institute (MNI) template brain using the standard SPM5 defaults. The functional images were smoothed with an isotropic 8-mm full-width at half-maximum Gaussian kernel. Finally, the value in each voxel was normalized by converting it into a percentage increase from the mean of the voxel in each session.

After the preprocessing, we fitted a linear regression (general linear) model to the data obtained from each participant (three sessions for Neymar and two sessions for the others). We prepared a separate regressor for each session. In each regressor, 8 runs (4 rightward and 4 leftward) were modeled with boxcar functions convolved with the canonical hemodynamic response function in SPM5. The regressor specified each run period with a hemodynamic delay in each session. Since Neymar had three sessions, we prepared three regressors for him and two for the others. We also included the head motion parameters estimated in the realignment procedure as regressors in each session to minimize the effects of the head motion artifacts. Finally, in the analysis, we removed the slow signal drifts (frequency > 128 s).

First, we depicted the brain regions where the BOLD signals increased during the foot movements by evaluating the beta values obtained from the two sessions (Figure 1; 2-session analysis). This was done separately for each participant to describe the general features of the individual brain activity during the foot movements. For Neymar, we used the data obtained from the first two sessions. In this 2-session analysis, we first generated an image of active voxels (cluster image) for each participant. To generate this image, consistently across participants, we used a voxel-wise threshold of *p* < 0.001 (uncorrected). Since our main interest was the motor-cortical foot regions, we set the region-of-interest (ROI) in the medial-wall foot motor regions (−20 < x < 0, −46 < y < 0, +40 < z in MNI coordinates). ROI was defined based on our previous study where we identified the foot sections in the human medial-wall motor regions (Naito et al., 2007). This ROI likely covers the supplementary motor area (SMA), the cingulate motor area (CMA), the primary motor cortex (M1), and the primary somatosensory cortex (SI) in the left hemisphere of an individual normalized brain. When we looked at the individual cluster image, we found that the largest cluster of brain activation was consistently located within this ROI across participants (Figure 1A). The medial-wall activation in each participant was significant at cluster-level (*p* < 0.05 corrected) when we evaluated its significance in terms of the spatial extent of activation within the ROI (Friston et al., 1994). We also counted the number of active voxels (voxel-wise threshold *p* < 0.001 uncorrected; Figure 1B) and identified their maximum *T*-values (Figure 1C) in each participant.

Next, we separately analyzed the increase of the BOLD signals during the foot movements for each session of each participant (single-session analysis). We also analyzed the data obtained from the third session for Neymar. We again consistently used the same voxel-wise threshold (*p* < 0.001 uncorrected) across the participants and counted the number of active voxels (Figure 2A) and identified their maximum *T*-values (Figure 2B) using ROI. We used this analysis result to evaluate the relationship between the brain activity and the foot movements (see below and Figure 2). The use of counting the number of active voxels as a measure of between-participant variability is still under debate. However, many studies suggests that this can be a good measure to evaluate individual difference (Binder et al., 1996; Carey et al., 2002; Brodtmann et al., 2007; Wang et al., 2012), though it might not be most reliable (Cohen and DuBois, 1999).

### Behavioral analysis

We performed offline analysis on the size of the foot movements using software (DIPP-Motion Pro2D, DITECT, Japan). After calibrating the sizes of the foot movements using the reference (bed width), we analyzed them in a two-dimensional plane perpendicular to the camera view. The location of the right big toe was plotted frame-by-frame (30 frames per second). Then we calculated the toe’s total movement distance in each run and separately averaged the movement distance for each session of each participant. This piece of data was used in the correlation analysis described above (Figure 2). In this analysis, we only quantified the size of the foot movements in the 2D plane; however, this still well described the size of the participant’s foot movements, since the main components were basically the rightward or leftward rotations in the 2D plane.

### Statistical evaluation

For the following statistical evaluation, we used statistical software (PASW Statistics 18, SPSS, Japan). In the 2-session analysis, we performed a nonparametric correlation analysis between the year of football experience and the data (number of active voxels and maximum *T*-value) across participants by calculating Spearman’s rank correlation coefficient.

In the single-session analysis, we performed a nonparametric Mann-Whitney test (two-tailed; Mann and Whitney, 1947) for the data (number of active voxels, maximum *T*-value, and average movement distance; Figure 2). First, we examined whether the data obtained from the professional footballers were significantly different from those obtained from the other participants (the swimmers and the amateur footballer; group analysis). In the analysis of each piece of data, the number of samples (*n*) was eight for the pro footballers and six for the other participants, i.e., two values for each participant.

Next, we examined whether the data obtained from a particular participant were significantly different from those obtained from the remaining participants (individual analysis). Here, *n* was 2 for a particular participant and 12 for the remaining 6 participants; we performed this individual analysis for all participants. In both the group and individual analyses, as for Neymar, we used the data obtained from the first two sessions. To validate the results of this individual analysis for Neymar’s data, we additionally performed an analysis that included the extra third session data (*n* = 3 for Neymar and 12 for the remaining 6 participants).

## Results

All the participants successfully performed their foot movements in synchronization with the 1-Hz metronome sounds. The mean movement distances across two sessions for the professional footballers ranged from 3.1 to 5.3 m and those obtained from the other participants (the swimmers and the amateur footballer) ranged from 3.3 to 8.5 m. As we describe the details of statistics below, no significant difference was observed between the two groups in terms of movement distance (see Section Results From Single-Session Analysis).

### Results from 2-session analysis

In this analysis, we depicted the general features of the individual brain activity during the foot movements and found that the movements activated the medial-wall motor regions in all the participants. The brain activity consistently formed the largest cluster in the entire brain across participants (Figure 1A). The number of active voxels in the medial-wall ROI (Figure 1B) was consistently smaller in the professional footballers (averaged 1092 voxels, ranging from 209 to 1866) than in the other participants (the swimmers and the amateur footballer; averaged 2384, ranging from 2070 to 2861). The number of active voxels (209) was smallest in Neymar’s brain (Figure 1B), and the maximum *T*-value in the medial-wall activity was also smallest in his brain (*T* = 5.08; Figure 1C). When we examined the relationship between the years of football experience and the number of active voxels across participants, we found a significant trend of negative correlation [Spearman’s *r* = −0.7, *N* = 7, *p* = 0.078 two-tailed (not shown in figure). Negative correlation (*r* = −0.3) was also observed between the year and the maximum *T*-value, but this did not reach significant level.

### Results from single-session analysis

In this analysis, we scrutinized the data for each session of each participant (Figure 2). First, we confirmed that the movement distance per session was similarly distributed across participants (horizontal axis in Figure 2), except the swimmer, YL (see green triangles in Figure 2 and below for the statistics). We also confirmed that both the number of active voxels and the maximum *T*-values had no clear relationship with the movement distance across participants (Figures 2A,B). The number of active voxels and the maximum *T*-values were relatively smaller in the pro footballers (squares in Figure 2) than in the other participants, as we observed in the 2-session analysis. This became apparent in Neymar (red filled squares in Figure 2).

The group analysis using the Mann-Whitney test showed that, despite no significant difference in the movement distance between the two groups (*p* = 0.18), the number of active voxels was significantly smaller in the pro footballers than in the other participants (*U* = 6, *p* < 0.05; squares in Figure 2A). The maximum *T*-values also showed a significantly smaller trend in the pro footballers (*U* = 9, *p* = 0.059; squares in Figure 2B).

As seen in Figure 2, the individual analysis using the Mann-Whitney test revealed that, only in Neymar, the number of active voxels was significantly smaller than in the remaining six participants (*U* = 1, *p* < 0.05; red filled squares in Figure 2A), with no difference in the movement distance from the others (*p* = 0.2). Neymar’s maximum *T*-values also showed a significantly smaller trend (*U* = 2, *p* = 0.088; see also the squares in Figure 2B).

Even when Neymar made larger movements in the extra third session (red open squares in Figure 2), his brain activity remained relatively small among the participants (active voxels = 466; maximum *T*-value = 5.46). This strongly indicates the consistency of our finding: smaller activity in Neymar’s medial-wall foot motor regions during foot movements. The additional individual analysis when we included his third session data showed that the number of active voxels and the maximum *T*-values were significantly smaller than those in the remaining six participants (*U* = 2, *p* < 0.05; *U* = 3, *p* < 0.05, respectively), with absolutely no difference in movement distance (*p* = 0.45). Thus, among all participants, the size of the medial-wall activity and its intensity were smallest in Neymar’s brain even though he generated comparable foot movements in size.

If we consider that much greater medial-wall activations were observed in participants SC, XB, MC, and SH, all of whom performed comparable movements in size (see the yellow, purple, blue, and navy symbols in Figure 2), the consistently smaller medial-wall activity in Neymar’s brain–even when he generated larger foot movements–may represent his individual quality in how he ordinarily uses his brain when controlling his foot movements.

The individual analysis using the Mann-Whitney test also showed that swimmer YL’s movement distance was greater than the other participants (*U* = 0, *p* < 0.05; see above) and that the number of active voxels and the maximum *T*-values in amateur SH were greater than those in the other participants (*U* = 0, *p* < 0.05 for both). This means that the amateur footballer recruited the greatest medial-wall activity among all the participants (see also Figure 1A panel SH).

Finally, we confirmed that the results for the number of active voxels in the single-session analysis are basically reproducible, even when we re-calculated the number of active voxels by adopting different voxel-wise thresholds ranged from *p* < 10^−7^ to 10^−1^.

## Discussion

In the present study, we successfully collected functional brain imaging data from Neymar da Silva Santos Júnior and compared his brain activity with that obtained from three other age-controlled professional footballers, two top-athlete swimmers and one amateur footballer in the same experimental environment (e.g., identical MRI scanner and image acquisition parameters). The relatively smaller number of participants and of experimental sessions and the lacking of complete non-athlete novice were the limitation of the current study. However, we found that, among all the present participants, the size of the medial-wall activity and its intensity were smallest in Neymar’s brain even though he generated comparable foot movements in size, which was consistently observed across experimental sessions (Figures 1, 2). Thus, we speculate that the smaller medial-wall activity during the foot movements is reliable and might reflect the characteristic use of the brain when he controls his foot.

The relatively small size of the medial-wall activations during foot movements in professional footballers (Figures 1, 2) seems to generally fit with previous findings in musicians (pianists, keyboard players and drummers), i.e., reduced recruitment of motor areas during finger movements compared with musically naïve control (Jäncke et al., 2000; Krings et al., 2000; Haslinger et al., 2004; Koeneke et al., 2004; Petrini et al., 2011). Perhaps intensive use of their feet through over-years training and experience that “manipulate” an external ball may cause long-term plastic changes in human central motor representation. Indeed, the significant trend of negative correlation between the year of football experience and the number of active medial-wall voxels suggests that the longer the football experience, the smaller the medial-wall activity in size. This could be true even though the present study did not systematically control the year of experience. We assume that the reduced BOLD effect in M1 is specific to an effector people are skilled at using. But at the present stage we only know a reduction in the hand section during finger movements in pianists (Krings et al., 2000) and a reduction in the foot section during foot movements in the present study.

Among the present professional footballers, Neymar’s medial-wall activation was the smallest both in size and strength (Figures 1, 2). Together with the correlation result, what we observed in Neymar’s brain might reflect a representative case of the long-term (over years) training effect in the medial-wall foot motor regions, though we cannot rule our other possibilities that there might be differences in type, quality and amount of current training between Neymar and other pro footballers. But Neymar seems to have richer sensory-motor experiences about foot movements because, since childhood, he claims to have used nearly 50 different types (size and quality of material) of balls and played with them barefoot. Such experiences could have shaped the characteristic use of his brain when controlling his foot.

Anyhow, we speculate how Neymar’s brain controls his foot movements based on recent findings in non-human primates (Picard et al., 2013). As briefly mentioned above, when monkeys perform acquired motor skills, a substantial reduction in the increase of metabolic activity reflecting synaptic activity can be observed in M1, while a single neuron firing is preserved. This can be interpreted as the over-years training of a motor skill that is likely to increase the synaptic efficacy in M1. If one supports the view that BOLD signals are well correlated with local field potential that reflects synaptic activity (Logothetis et al., 2001), the lesser BOLD increase in Neymar’s medial-wall foot regions (Figures 1, 2) implies that synaptic efficacy increases in these motor regions. If this is the case, since the signal-transmission efficacy should increase in the motor-cortical synapses, motor-cortical cells can fire without receiving massive synaptic inputs. Another possibility is that Neymar’s brain recruits only the limited sections in the medial-wall motor regions when he controls the simple foot movements used in this study. If this is true, lesser cells including cortico-motoneuronal cells (sending motor commands to muscles via spinal motoneurons) need to be recruited to control foot movements. Both possibilities are likely associated with the following two factors: (1) reduction of signal-dependent noise on motor commands in the control of foot movements, which is normally the neural source to generate variability in a performed motor skill (Churchland et al., 2006); and (2) very efficient motor control (presumably highly sophisticated muscle synergy control) with less effort is necessary for foot movements *per se*.

Neymar may efficiently control foot movements by largely conserving motor-cortical neural resources probably with higher reproducibility and less effort. Ideally, a greater range of foot movements should be assessed to further substantiate this conclusion. It is intriguing to speculate that conserving motor-cortical resources when performing simple foot movement may in turn expand the possible control capacity for a wide range of football skills since the remaining resources can be assigned to control a variety of movements of lower extremities. We may also assume that this fundamental capability of his football brain could allow him to spend neural resources to focus more on cognitive aspects during a football game, such as anticipating/predicting and detecting the actions of other players (cf. Wright et al., 2013). It might be worth testing in future study whether or not reduced recruitment of foot motor regions in football expert’s brain is associated with multi-tasking ability when they perform a cognitive task during foot movements.

## Conflict of interest statement

The authors declare that the research was conducted in the absence of any commercial or financial relationships that could be construed as a potential conflict of interest.
